# Coronavirus epidemic in Croatia: case fatality decline during summer?

**DOI:** 10.3325/cmj.2020.61.501

**Published:** 2020-12

**Authors:** Ivica Kristić, Marina Pehlić, Mirjana Pavlović, Branko Kolarić, Ivana Kolčić, Ozren Polašek

**Affiliations:** 1 Department of Cardiology, University Hospital Split, Split, Croatia; 2 Department of Gynecology, University Hospital Split, Split, Croatia; 3 Clinic for Tumors, Sestre Milosrdnice University Hospital, Zagreb, Croatia; 4 Department of Social Medicine and Epidemiology, Medical Faculty, University of Rijeka, Rijeka, Croatia; 5 Andrija Štampar Teaching Institute of Public Health, Zagreb, Croatia; 6 Department of Public Health, University of Split School of Medicine, Split, Croatia; * The first two authors contributed equally.

## Abstract

**Aim:**

To describe the SARS-CoV-2 epidemic pattern in Croatia during February-September 2020 and compare the case fatality ratio (CFR) between spring and summer.

**Methods:**

National data were used to calculate the weekly and monthly CFRs, stratified by three age groups: 0-64, 65-79, and 80+ years. We also calculated the standardized mortality ratios (SMR) to offset the differences in age composition.

**Results:**

The epidemic consisted of the initial wave, a trough in June, and two conjoined summer waves, yielding 17 206 coronavirus disease 2019 cases and 290 deaths. While the number of confirmed cases nearly quadrupled during summer, case fatality estimates decreased; CFR in spring was 4.81 (95% confidence interval 3.91-5.71), compared with 1.24 (1.06-1.42) in summer. The SMR for summer was 0.45 (0.37-0.55), suggesting that the case fatality risk halved compared with spring. Cardiovascular comorbidity was an important risk factor for case fatality (SMR 2.63 [2.20-3.13] during spring and 1.28 [1.02-1.59] during summer). The risk of death in ventilated patients remained unchanged (SMR 0.98 [0.77-1.24]).

**Conclusions:**

The epidemic dynamics suggests summer decline in case fatality, except in ventilated patients. While the effect of comorbidity also decreased, cardiovascular comorbidity remained an important risk factor for death even during summer. A plethora of possible confounders and an ever-changing landscape of SARS-CoV-2 epidemic in Croatia require constant monitoring and evaluation, with an aim to prevent the uncontrolled spread of the virus and a disruption of health care functioning.

The understanding of the seasonal pattern of SARS-CoV-2 spread and coronavirus disease 2019 (COVID-19) symptoms severity is not of only academic significance, but plays a central role in the epidemic preparedness (1-4). For the Northern hemisphere, the issues currently of the greatest importance are the prediction of the autumn and winter disease spread and mortality (5). Previous articles have most commonly confirmed the expected role of seasonality (6), often with an explanation that higher temperature reduces the virus spread during summer (7-9). On the other hand, some studies have shown that we may not expect a substantial seasonality pattern, suggesting that SARS-CoV-2 may not behave like a common seasonal respiratory pathogen (10-12). A recent systematic review confirmed the existence of some degree of seasonality, but also stated that it may not be the only or the critical variable that will define the pandemic patterns (13).

There are various possible causes underlying the seasonal pattern of the pandemic, including changes in temperature and humidity (14-17), or wind speed and air pollution (18). Additionally, unfavorable conditions were reported to affect the ability of respiratory mucosa to fend off (19), which suggests a possible synergistic effect between numerous factors. An important distinction needs to be made by separately addressing the viral spread and case fatality, since the two might not exhibit similar patterns. The recognition of such differences may shed more light on the disease pathogenesis, and possibly even steer the path toward mechanisms of epidemic control. However, studies that made this distinction often report the expectation that for Europe, an increase in humidity will lower the number of new cases and deaths, while a rise in temperature will increase these parameters (20). Although such predictions are very sensitive to numerous modeling input parameters and assumptions, none can be superior to the epidemiological *ex post-facto* analysis. Several such articles suggested lowering of the summer case fatality (9,21), but this finding was not replicated systematically across all studies (22). Therefore, the aim of this study was to explore the epidemic pattern in Croatia, with a special focus on understanding the difference in the disease spread and case fatality during the spring and summer of 2020.

## Material and methods

The main source of information were the databases of the Croatian Institute of Public Health and the Croatian National Health Insurance Fund. The final data set encompassed the data from the period February-September 2020 (ending with the data available on September 09, 2020), accompanied by the available information on the testing outcomes, disease progression, the use of hospital services, and the disease outcome. In order to obtain the comorbidity information, we merged the data set with the Croatian National Health Insurance usage data from the previous three years. For this period, we defined all the ICD-10 disease categories attributed to a single patient, and used this as the information on comorbidity status. In order to perform a pooled analysis, we classified all comorbid conditions by the first letter of the ICD-10 classification code, meaning that only the disease category was used as comorbidity information (since we had no means of harmonizing the data to obtain more refined estimates). All of the comorbidity categories were scored as binary variables, allowing us to calculate the overall comorbidity load, defined as the sum of all positive results of comorbid conditions. This variable was only assumed as a relative proxy of the comorbidity status, as it suffers from numerous methodological risks and biases; it was based only on registered and diagnosed conditions, each ICD-10 category was treated as having equal weight, the disease severity or duration was not estimated, to name just the most obvious ones. In addition, we used three more binary comorbidity-related variables: the presence of any comorbidity, cardiovascular comorbidity, or tumor-related comorbidity.

All the data were sorted according to the epidemic duration into weeks and months, enabling us to analyze the selected parameters as the epidemic progressed. Notably, the data for September were included without the ability to infer the outcomes for some patients, therefore any estimates from September, especially those related to case fatality, are only treated as preliminary and provisional. Two main analytic periods were loosely defined as spring (February to May) and summer (June to September). Although this breakdown does not strictly conform to the official start and end of the seasons, we considered the analyzed periods to be good proxies for spring and summer.

All the data were routinely collected and anonymized at the source, before the export to the analytic file was made. No personal information data were available to the authors during any part of the study execution; therefore, no ethical approval for this study was sought.

### Statistical analysis

The principal focus of the study was the calculation of the case fatality ratio (CFR), defined as the number of the deceased among the total number of confirmed cases. The CFRs were calculated for every week and month of the epidemic, as well as for spring vs summer. However, for the purposes of this study, we used the diagnosis date as the reference point, regardless of disease duration and outcome. In effect, this meant that every case of death was scaled according to the date of diagnosis, in order to provide a better estimate of seasonality and offset the differences in disease duration (which sometimes extended for longer than a month). This also meant that the CFRs were less biased, since the disease duration effect was counteracted (as opposed to the aggregated intra-epidemic daily CFRs, which suffer from bias due to the outcome lag). For every point estimate of CFR, we also calculated the 95% confidence intervals.

In order to offset the differences in the age composition between units of observation, we calculated the standardized mortality ratios (SMR). For this purpose, we used the five-year breakdown according to age, with spring as the referent and summer as the target group; this meant that the SMRs reflected the age-adjusted spring vs summer differences. We also used SMRs in several more situations where comparisons were assumed to be confounded by the age structure.

The calculation of the delay from diagnosis to hospitalization was restricted to the period of 0-7 days, in order to remove possible bias due to other causes of hospitalization in previously positive patients. For a few deceased patients in whom the disease duration was not evident, as the disease duration indicator we used the time from diagnosis to death.

Categorical data were analyzed with the χ^2^ test, correlations with the Pearson correlation test, while numerical data were analyzed with ANOVA or the *t* test, depending on the number of analyzed categories. We also used logistic regression for case fatality prediction, with age, sex, season, and comorbidity load as covariates. All the data were analyzed with R, with significance level set at *P* < 0.05.

## Results

**Figure 1:**
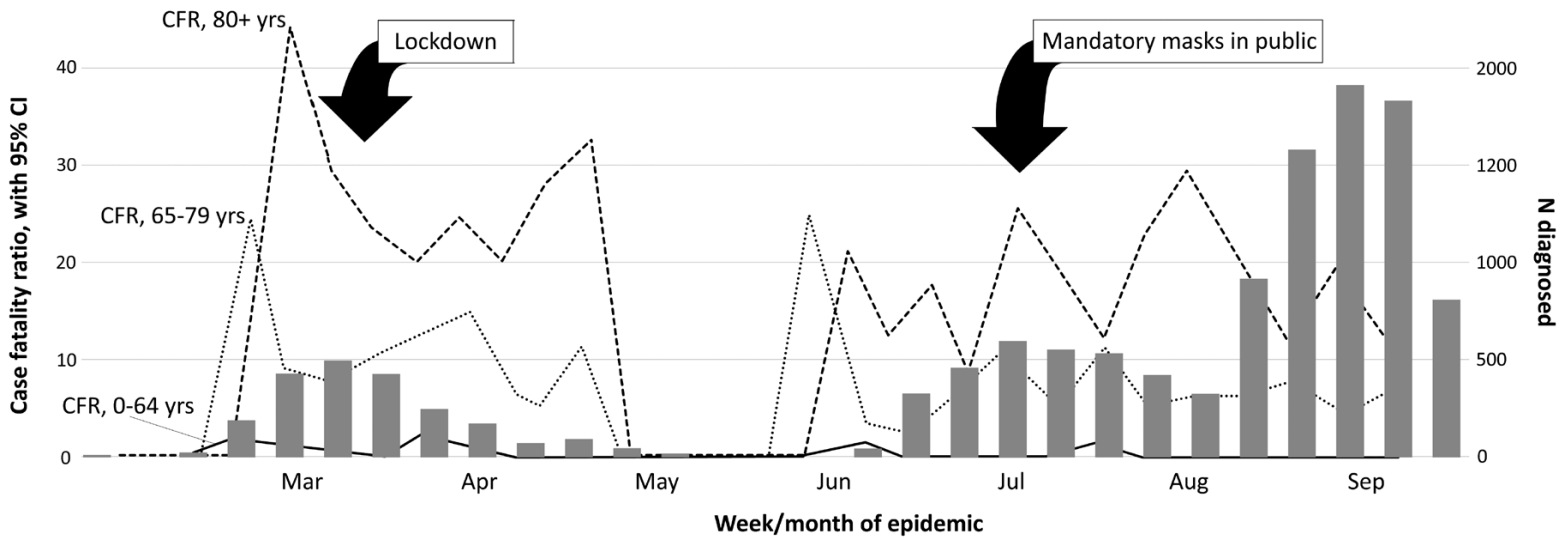
Comprehensive scheme of the SARS-CoV-2 epidemic in Croatia. The left axis denotes the case fatality ratio (CFR), with cases of deaths being classified according to the date of diagnosis (mortality lag removed). The right axis denotes the daily number of newly diagnosed cases (gray bars). Case fatality ratios were stratified to three age groups (0-64 years, 65-79 years, ≥80 years old).

From the beginning of the epidemic to the end of the study period, there were 17 206 cases of COVID-19 in Croatia, distributed in three outbreaks: the first corresponded to spring, while the second and third were overlapping in summer (Figure 1, right axis; Supplementary Table 1(supplementary table 1)). The epidemic pattern was marked with several important events, namely the lockdown, including school closings and a ban on inter-city travel (March 13-23), and the decision on the mandatory use of masks in public areas and transport (July 13). Additionally, there was a three-week period in June when the newly diagnosed cases and deaths neared zero (Figure 1). During the entire epidemic, the case fatality ratios varied, with the highest values among the oldest patients, and lower values during the summer months (Figure 1, left axis; Supplementary Table 1(supplementary table 1)). ). The overall case fatality for spring was 4.8% (108 cases), while that for summer was 1.2% (182 cases; *P* < 0.001). Notably, the CFRs exhibited the highest decline in summer in the youngest age group and the lowest in the oldest age group (Supplementary Table 2(supplementary table 2)). ).

The standardized mortality ratio confirmed a reduction in case fatality, with SMR = 0.45 (95% CI 0.37-0.55]), which was similar in men (0.45 [0.34-0.58]) and women (0.45 [0.32-0.60]). The presence of any kind of comorbid conditions nearly doubled the SMR in spring (2.02 [1.66-2.44]), while it decreased to having no effect in summer (1.00 [0.46-1.89]). The SMRs for cardiovascular diseases decreased substantially (2.63 [2.20-3.13] in spring vs 1.28 [1.02-1.59] in summer). On the contrary, the SMR for tumors was 1.12 [0.83-1.48] in spring, while in summer it seemed to have a somewhat protective effect, with a SMR of 0.45 (0.36-0.56). Finally, the logistic regression model that investigated the risk factors for death outcome suggested that female sex and summer infection were protective, while older age and accumulating comorbidity were detrimental effects; notably, the odds ratio for the oldest age group was substantial (Table 1).

**Table 1:** Predictors of death outcomes of coronavirus 2019 in the Croatian population from March to September 2020, logistic regression

Predictor	Odds ratio [95% confidence interval]	*P*
Sex		
male (Ref.)	1.00	<0.001
female	0.51 [0.39-0.66]	
Age (years)		
0-64 (Ref.)	1.00	<0.001
65-79	19.6 [13.6-28.4]	
80 and more	58.2 [39.5-85.8]	
Comorbidity load	1.07 [1.03-1.11]	0.001
Season		
spring (Ref.)	1.00	<0.001
summer	0.50 [0.38-0.66]	

There were 57 patients requiring assisted ventilation in spring (2.5% out of confirmed cases), compared with 96 patients (0.6%) in summer (*P* < 0.001). The SMR for summer vs spring suggested that the ratio for ventilator use more than halved (0.41 [0.33-0.50]). However, the progression from ventilation to death did not show significant differences between the seasons; 37 (65%) of ventilated patients deceased in spring, compared with 70 (73%) in summer (*P* = 0.297). Accordingly, the SMR for dying after the ventilation use did not differ between summer and spring (0.98 [0.77-1.24]).

Delay of hospitalization from the time of diagnosis increased during the observed period, from 1.00 ± 0.82 days in February, 1.17 ± 1.40 in March, 0.93 ± 1.14 in April, 0.85 ± 0.68 in May, 1.94 ± 2.13 in June, 1.85 ± 2.18 in June, 2.20 ± 2.19 in August to 2.31 ± 2.36 in September (monthly comparison ANOVA *P* < 0.001; spring vs summer *P* < 0.001). Even the percent of hospitalized patients was different; there were 886 hospitalized cases in spring (39.3%), compared with 1784 in summer (11.9%; *P* < 0.001).

Duration of hospital treatment declined significantly as the epidemic progressed, from 22.8 ± 11.8 days in February to 7.6 ± 6.0 days in September (*P* < 0.001); the duration of hospital treatment did not show any coherent pattern in regards to the disease outcome (Supplementary Table 3(supplementary table 3)). ). Duration of the hospital stay was the longest in the oldest age group and the shortest in the youngest age group (80+ years old: 16.6 ± 11.0 days; 64-79 years old: 13.7 ± 10.8; 0-64 years old: 9.4 ± 8.0 days). The comorbidity load was significantly associated with the duration of hospital stay (*P* < 0.001; r = 0.16).

## Discussion

This is the first systematic attempt to describe the dynamics and outcome of the first eight months of the SARS-CoV-2 epidemic in Croatia and compare the mortality patterns in spring and summer. While the number of newly diagnosed cases increased substantially during summer, the CFR declined. The summer case fatality was nearly halved in comparison with the spring rate, with the greatest reduction observed in the youngest age group. Additionally, the presence of any comorbidity doubled the risk of dying in spring, while the risk declined to a no detectable effect in summer. The exception was cardiovascular disease, which retained its detrimental effect. This suggests that either comorbidity could only be a conditional risk factor that operates in unfavorable conditions or that comorbidity had an additive detrimental effect in spring, which was dissolved in summer. Unfortunately, previously published papers provide little evidence as to the extent of comorbidity contribution, which ranges from substantial (23) to none (24), and any level of contribution in between.

The summer case fatality rates decline could have been caused by a number of factors, broadly classifiable into pathogen-related, host-related, or environmental (and any combination or interaction thereof). Enveloped viruses are known to have stronger seasonal occurrence (25,26), possibly due to the lipid (27) or glycosylation properties of their envelope (28,29). On the other hand, human mucosa also shows seasonal changes, probably affecting mucosal barrier and clearance mechanisms, as a response to the environmental conditions (17,30). Respiratory tract microbiome was also shown to exhibit seasonal patterns (31,32), possibly attributing to the seasonal pattern of respiratory infections. Interestingly, challenge studies of cold air inhalation have failed to replicate the seasonal effects (33-35), suggesting that the exact pathogenetic mechanisms of increased winter-spring burden of respiratory pathogens are very complex. Therefore, the lack of clarity and understanding of causal relationships at this stage reduce the ability to use seasonal effects mechanisms as the COVID-19 epidemic controlling measures.

Cardiovascular comorbidity seemed to have a strong modifying effect, which persisted throughout both spring and summer. Previous studies have already established this link (36-38), reporting a strong modifying effect on the COVID-19 prognosis (39-42). However, studies have reported not only that pre-existing cardiovascular comorbidity affects the prognosis, but also that SARS-CoV-2 may trigger myocardial injury, acute coronary syndrome, arrhythmias, and venous thromboembolism (39,43-45). As a result, COVID-19 patients often have the signs of myocardial injury (39,46-48), with more than half of all COVID-related deaths exhibiting signs of heart failure (49). A large study from Italy suggested that none of the comorbid conditions increased the mortality risk, but that the risk was conveyed by the impaired renal function, elevated C-reactive protein, and advanced age (50). Interestingly, in our study the detrimental effects of tumor comorbidity were dissolved in summer, conveying even a significant reduction of case fatality risk. Similar results were already reported (51), suggesting that cancer effects on the risk of dying in COVID-19 patients might even be case-, stage-, or grade-specific. Alternatively, patients with cancer might have more strictly adhered to preventative measures, which led to a lower risk of dying in summer. All these results suggest that a proper answer to the question of comorbidity contribution will have to be given in a form of a systematic review and meta-analysis, preferably using individual-level data (52).

The data presented here also suggest that clinical approach to patients with COVID-19 has likely changed. Hospitalization due to COVID-19 during summer was three times as less likely as it was during spring. While the duration of the hospital stay declined, the delay from diagnosis to hospitalization was prolonged. This means that we no longer hospitalize all the patients, and that we possibly have better treatment outcomes, which are reflected in a shorter stay. This confirms the complexity of the COVID-19 management within the health care system (53).

This study has numerous limitations. The quality of the input data had not been assessed or validated. Further, the data collection process was not harmonized or created for this purpose, especially early on during the epidemic course, which could be a major source of risk of bias and error. Next, heterogeneity of treatment plans across different hospitals and across the epidemic duration, including diagnosis criteria, severity assessment, hospital admission, or ventilator use were changing, meaning that their direct pooling may yield spurious results. This epidemic has produced a substantial societal effect, meaning that the epidemic landscape might change quickly, as it has in the past, making all these estimates time-sensitive. The changing capacity and pattern of testing could have also affected the results, possibly by underestimating the real number of cases (54,55). The extent and direction of this is hard to estimate; low testing may underestimate the true number of positive results, while selective under-testing (and consequent under-diagnosing) of certain population groups could have substantially skewed the results; this is why the improvement of testing capacities is a critical step forward (56). Finally, the methodological framework that uses CFR and SMR is also a possible source of problems, including the analysis of the data from an ongoing epidemic, which reduced the usability of estimates toward the end of observation period.

The results of this study demonstrate the apparent changes in the outcomes during summer, which systematically suggest favorable direction and confirm previous predictions of the virus and epidemic seasonality. Therefore, this study provides an account of the mortality patterns, ultimately aiming to contribute to a better epidemic management and the prevention of health care functioning disruptions.
