# Supplementary material for: Coronavirus epidemic in Croatia: case fatality decline during summer?
Source: Croat Med J. 2020 Dec;61(6):501–7. doi: 10.3325/cmj.2020.61.501 (PMC7821368; doi:10.3325/cmj.2020.61.501)
Supplement: Supplementary Table 1 [file CroatMedJ_61_s004.pdf]

Supplementary Table 1. Number of deceased and registered cases, case fatality ratios and 95% confidence intervals for three age groups

	0-64 years		65-79 years		80 and more years	
Week[a]	Deceased/total	CFR [95% CI]	Deceased/total	CFR [95% CI]	Deceased/total	CFR [95% CI]
1 [21-FEB-2020]	0/5	0 [0-0]	0	0	0	0
2 [28-FEB-2020]	0/6	0 [0-0]	0	0	0	0
3 [06-MAR-2020]	0/19	0 [0-0]	0/1	0 [0-0]	0	0
4 [13-MAR-2020]	2/100	2.00 [-0.8-4.8]	2/8	25.00 [-5.62-55.62]	0/4	0 [0-0]
5 [20-MAR-2020]	5/369	1.36 [0.16-2.56]	7/78	8.98 [2.51-15.45]	4/9	44.45 [11.32-77.58]
6 [27-MAR-2020]	3/409	0.74 [-0.12-1.58]	6/78	7.70 [1.66-13.73]	8/28	28.58 [11.50-45.65]
7 [03-APR-2020]	1/291	0.35 [-0.35-1.03]	8/76	10.53 [3.49-17.57]	13/56	23.22 [11.94-34.50]
8 [10-APR-2020]	4/189	2.12 [0.03-4.22]	5/39	12.83 [2.12-23.53]	12/60	20.00 [9.68-30.33]
9 [17-APR-2020]	1/108	0.93 [-0.92-2.77]	5/33	15.16 [2.67-27.64]	12/48	25.00 [12.50-37.50]
10 [24-APR-2020]	0/56	0 [0-0]	1/14	7.15 [-6.63-20.91]	1/5	20.00 [-15.78-55.78]
11 [01-MAY-2020]	0/55	0 [0-0]	1/15	6.67 [-6.22-19.55]	4/14	28.58 [4.43-52.72]
12 [08-MAY-2020]	0/38	0 [0-0]	1/8	12.5 [-10.89-35.89]	2/6	33.34 [-5.16-71.83]
13 [15-MAY-2020]	0/14	0 [0-0]	0/4	0 [0-0]	0/3	0 [0-0]
14 [23-MAY-2020]	0/2	0 [0-0]	0	0	0/1	0 [0-0]
15 [03-JUN-2020]	0/1	0 [0-0]	0	0	0	0
16 [09-JUN-2020]	0/2	0 [0-0]	0	0	0	0
17 [12-JUN-2020]	0/28	0 [0-0]	1/4	25.00 [-18.31-68.31]	0	0
18 [19-JUN-2020]	1/210	0.48 [-0.48-1.43]	5/41	12.20 [1.98-22.42]	3/14	21.43 [-0.51-43.37]
19 [26-JUN-2020]	1/423	0.24 [-0.24-0.71]	1/36	2.78 [-2.71-8.26]	1/8	12.50 [-10.89-35.89]
20 [03-JUL-2020]	2/481	0.42 [-0.18-1.01]	2/60	3.34 [-1.31-7.97]	3/17	17.65 [-0.85-36.14]
21 [10-JUL-2020]	1/451	0.23 [-0.23-0.67]	8/73	10.96 [3.65-18.28]	3/35	8.58 [-0.90-18.04]
22 [17-JUL-2020]	2/470	0.43 [-0.18-1.03]	7/78	8.98 [2.51-15.45]	7/27	25.93 [9.06-42.80]
23 [24-JUL-2020]	1/373	0.27 [-0.27-0.81]	2/47	4.26 [-1.64-10.15]	3/17	17.65 [-0.85-36.14]
24 [31-JUL-2020]	2/253	0.80 [-0.33-1.91]	5/38	13.16 [2.2-24.13]	3/28	10.72 [-0.98-22.41]
25 [07-AUG-2020]	2/763	0.27 [-0.11-0.64]	1/22	4.55 [-4.34-13.43]	3/13	23.08 [-0.30-46.45]
26 [14-AUG-2020]	0/1439	0 [0-0]	4/69	5.80 [0.18-11.43]	7/23	30.44 [11.25-49.63]

27 [21-AUG-2020]	5/1594	0.32 [0.04-0.60]	7/125	5.60 [1.49-9.72]	9/40	22.50 [9.30-35.71]
28 [28-AUG-2020]	3/1692	0.18 [-0.03-0.39]	14/205	6.83 [3.31-10.36]	8/63	12.70 [4.31-21.09]
29 [04-SEP-2020][b]	5/1390	0.36 [0.04-0.69]	9/180	5.00 [1.76-8.25]	10/51	19.61 [8.49-30.73]
30 [11-SEP-2020][b]	1/1211	0.09 [-0.09-0.25]	10/158	6.33 [2.46-10.21]	5/37	13.52 [2.28-24.76]
31 [18-SEP-2020][b]	0/1056	0 [0-0]	4/167	2.40 [0.03-4.77]	8/84	9.53 [3.12-15.93]
32 [25-SEP-2020][b]	0/1131	0 [0-0]	1/163	0.62 [-0.61-1.84]	2/49	4.09 [-1.58-9.74]
33 [02-OCT-2020][b]	0/14	0 [0-0]	0/2	0 [0-0]	0/1	0 [0-0]
Total	42/14.643	0.29 [0.20-0.38]	117/1822	6.43 [5.28-7.58]	131/741	17.68 [14.88-20.49]

a - Starting date for every week; b – biased estimates, due to ongoing epidemic
