# Supplementary material for: Coronavirus epidemic in Croatia: case fatality decline during summer?
Source: Croat Med J. 2020 Dec;61(6):501–7. doi: 10.3325/cmj.2020.61.501 (PMC7821368; doi:10.3325/cmj.2020.61.501)
Supplement: Supplementary Table 2 [file CroatMedJ_61_s005.pdf]

Supplementary Table 2. Case fatality ration for three age groups during spring and summer

Age group	Spring		Summer	
	Deceased/ total	CFR	Deceased/ total	CFR
0-64 years	16/1661	0.97 [0.49-1.45]	26/12982	0.21 [0.13-0.28]
65-79 years	36/354	10.17 [6.96-13.39]	81/1468	5.52 [4.33-6.71]
80 and more years	56/234	23.94 [18.36-29.52]	75/507	14.8 [11.64-17.95]
