# Supplementary material for: Coronavirus epidemic in Croatia: case fatality decline during summer?
Source: Croat Med J. 2020 Dec;61(6):501–7. doi: 10.3325/cmj.2020.61.501 (PMC7821368; doi:10.3325/cmj.2020.61.501)
Supplement: Supplementary Table 3 [file CroatMedJ_61_s006.pdf]

Supplementary Table 3. Duration of hospital treatment of survived and deceased patients

	Duration of hospital treatment			
Month	Total	Survived	Deceased	P
February	22.8±11.8	22.83±11.79	-	-
March	14.9±9.8	14.57±9.78	18.97±9.54	0.018
April	17.1±11.3	17.61±10.9	14.33±12.77	0.028
May	13.4±10.3	13.76±10.49	11.38±9.37	0.551
June	12.2±12.0	11.83±12.35	16.0±6.31	0.320
July	10.8±9.3	10.26±8.81	16.5±13.01	<0.001
August	9.4±8.4	9.22±8.42	11.38±8.26	0.121
September[a]	7.6±6.0	7.51±6.02	9.56±4.08	0.177

a - biased estimates due to ongoing epidemic
